# Quantitative multiplex detection of plant pathogens using a novel ligation probe-based system coupled with universal, high-throughput real-time PCR on OpenArrays™

**DOI:** 10.1186/1471-2164-8-276

**Published:** 2007-08-14

**Authors:** Ronald van Doorn, Marianna Szemes, Peter Bonants, George A Kowalchuk, Joana F Salles, Elen Ortenberg, Cor D Schoen

**Affiliations:** 1 Plant Research International B.V., Droevendaalsesteeg 1, 6708 PB, Wageningen, the Netherlands; 2 NIOO-Centre for Terrestrial Ecology, P.O. Box 40, 6666 ZG, Heteren, the Netherlands; 3 University of Bristol, Department of Cellular and Molecular Medicine, University Walk, Bristol, BS8 1TD, UK; 4 Free University of Amsterdam, Institute of Ecological Science, De Boelelaan 1085, 1081 HV, Amsterdam, the Netherlands; 5 UMR CNRS 5557- Université Lyon 1, USC INRA 1193, Microbial Ecology Centre, F-69622 Villeurbanne, France; 6 BioTrove, Inc. 12 Gill Street, Woburn, MA 01801-1728, USA

## Abstract

**Background:**

Diagnostics and disease-management strategies require technologies to enable the simultaneous detection and quantification of a wide range of pathogenic microorganisms. Most multiplex, quantitative detection methods available suffer from compromises between the level of multiplexing, throughput and accuracy of quantification. Here, we demonstrate the efficacy of a novel, high-throughput, ligation-based assay for simultaneous quantitative detection of multiple plant pathogens. The ligation probes, designated Plant Research International-lock probes (PRI-lock probes), are long oligonucleotides with target complementary regions at their 5' and 3' ends. Upon perfect target hybridization, the PRI-lock probes are circularized via enzymatic ligation, subsequently serving as template for individual, standardized amplification via unique probe-specific primers. Adaptation to OpenArrays™, which can accommodate up to 3072 33 nl PCR amplifications, allowed high-throughput real-time quantification. The assay combines the multiplex capabilities and specificity of ligation reactions with high-throughput real-time PCR in the OpenArray™, resulting in a flexible, quantitative multiplex diagnostic system.

**Results:**

The performance of the PRI-lock detection system was demonstrated using 13 probes targeting several significant plant pathogens at different taxonomic levels. All probes specifically detected their corresponding targets and provided perfect discrimination against non-target organisms with very similar ligation target sites. The nucleic acid targets could be reliably quantified over 5 orders of magnitude with a dynamic detection range of more than 10^4^. Pathogen quantification was equally robust in single target versus mixed target assays.

**Conclusion:**

This novel assay enables very specific, high-throughput, quantitative detection of multiple pathogens over a wide range of target concentrations and should be easily adaptable for versatile diagnostic purposes.

## Background

There is an ever-increasing demand for high-throughput, standardized assays for the quantitative detection of multiple infectious agents in different disciplines, ranging from clinical diagnostics, food safety, homeland security, and environmental pest management to the evaluation of treatment efficacy [1-4].

Current technologies for multiplex, quantitative analyses frequently suffer from compromises between the level of multiplexing, throughput and accuracy of quantification. In general, for the detection of nucleic acids, microarrays and macroarrays, can provide very high levels of multiplexing [5,6], but yield a limited range of accurate quantitative information [7] and relatively low sample throughput compared to real-time, quantitative PCR (qPCR) [8,9]. At present, qPCR provides the most reliable means of target quantification, and it is suitable for the analysis of relatively large sample numbers [10,11]. Nowadays, successful multiplex qPCR-based pathogen detection methodologies have been realized [12-14]; however, due to a limited number of potential dyes, the attainable level of multiplexing is low [9,15]. Moreover, the accuracy of quantification in samples with unbalanced target ratios is limited [16-18]. To guarantee accurate quantification therefore, single targets with internal controls should be analyzed, which results in a dramatic increase in the number of reactions needed to be performed in large scale screenings.

**Figure 1:**
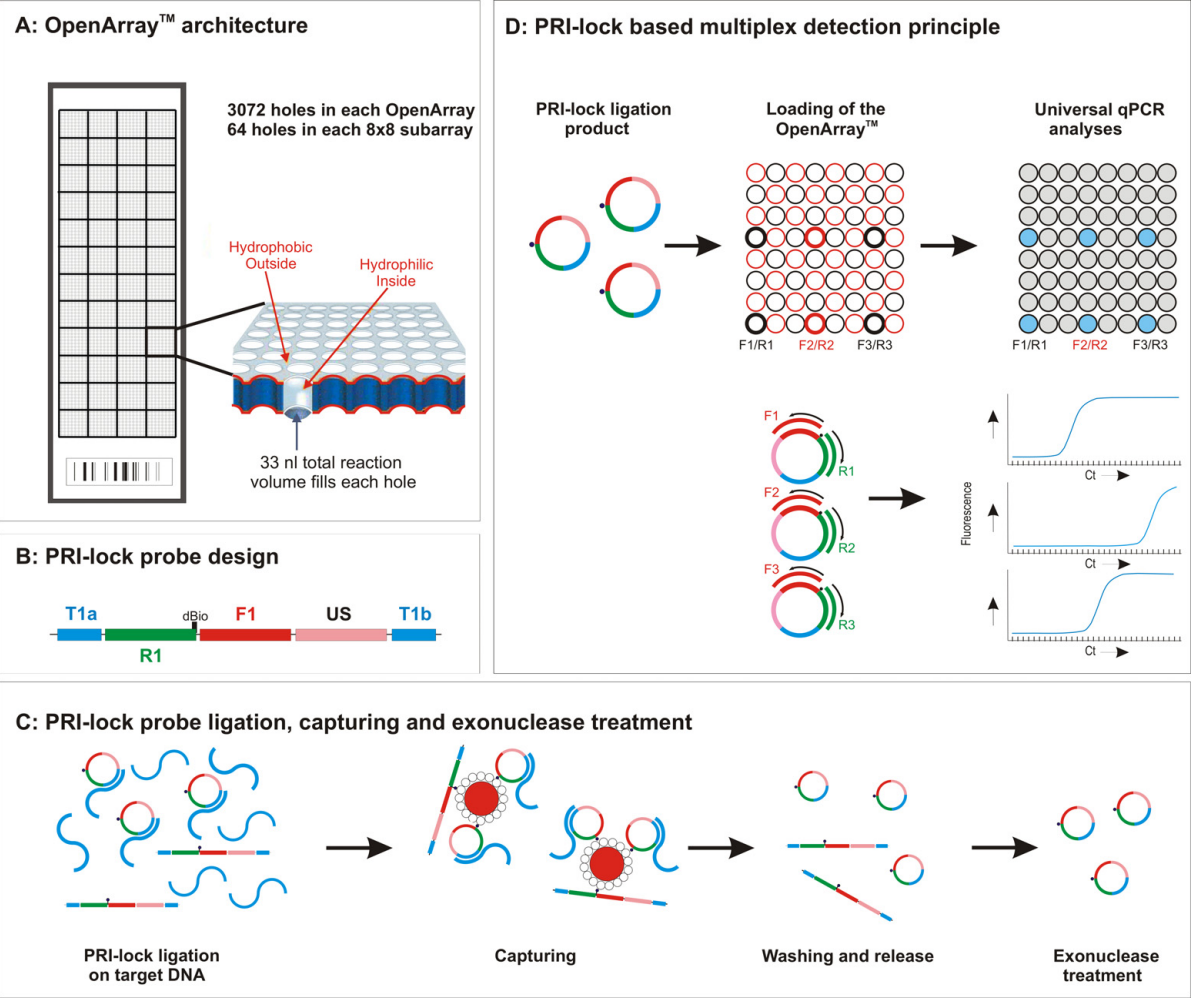
Schematic overview of the proposed assay. (**A**) OpenArray™ architecture. The OpenArray™ has 48 subarrays and each subarray contains 64 microscopic through-holes of 33 nl volume. The primers are pre-loaded into the holes. The sample combined with the reaction mix is auto-loaded due to the surface tension, provided by the hydrophilic coating of the holes and the hydrophobic surface of the array. (**B**) PRI-lock probe design. T1a and T1b indicate target complementary regions. Unique primer sites ensure specific amplification (forward: F1 and reverse: R1) and each PRI-lock contains a universal sequence (US) and a desthiobiotin moiety (dBio). (**C**) Multiple target specific PRI-lock probes are ligated on fragmented DNA samples. T1a and T1b bind to adjacent sequences of the target and in case of a perfect match, the probe is circularized by a ligase. The probes are captured via the desthiobiotin moiety using magnetic streptavidin-coated beads. The PRI-lock probes are washed and quantitatively eluted from the beads. Unreacted probes are removed by exonuclease treatment. (**D**) Circularized probes are loaded and independently amplified on the Biotrove OpenArray™ platform using PRI-lock probe specific primers. The amplification is monitored using SYBR-Green and the ligated PRI-lock probes are quantified based on the threshold cycle number (C_T_).

Recently, a conceptually new, high-throughput platform has become available for real-time PCR, capable of accommodating more than 3000 reactions per array [19]. The OpenArray™ has 48 subarrays, allowing parallel testing of up to 48 samples, and each subarray contains 64 microscopic through-holes of 33 nl volumes (Figure 1A). Primers are pre-loaded and dried down in user specified holes. The holes function as capillaries, accurately self metering sample and master mix added by an automated loader. Hydrophilic holes and hydrophobic array-surface coatings ensure the sample remains isolated through surface tension.

Pre-formatted, high-throughput assays are very useful in several fields of clinical and basic research. They lack, however, the necessary flexibility and adaptability to address rapidly changing needs in various diagnostic and ecological fields. Further, the needs of these fields are rarely addressed by pre-developed and commercially available assays. Therefore, the possibility to develop new applications for a universal, high-throughput real-time PCR platform would be a significant advancement as it increases user flexibility and lowers development cost.

There are a number of hurdles that have hampered the development of efficient and reliable multiplex detection systems. These include the need for high levels of sensitivity as well as specificity, coupled with quantitative, multiplex capabilities and high sample throughput. The assay system described in this report incorporates the most attractive features of several approaches to provide a unique detection platform that combines multiplex technology with stringent diagnostic standards: (1) high specificity is achieved via the use of ligation-based circularization probes, (2) real-time PCR methodology provides excellent sensitivity and accurate quantification, and (3) adaptation to the newly developed OpenArray™ technology facilitates high-throughput sample screening.

Here, we propose that a new, ligation based probe assay can bridge the gap between highly specific target recognition and high-throughput, multiplex pathogen quantification. Circularizable ligation probes (Padlock probes) [20] have previously been applied successfully for multiplex pathogen detection [21], but do not allow quantification. In the currently developed assay, the circularized probes are amplified by using probe-unique primer pairs via real-time PCR, enabling accurate target quantification in a highly multiplex format. The utilized probes have been termed PRI-lock probes and they consist of two target complementary regions, one at each terminus of the probe (Figure 1B). The target complementary arms are connected via a compound linker sequence, containing unique primer binding sites, a generic sequence and a desthiobiotin moiety for specific capture and release [22] (Figure 1B). The primer binding sites were selected to ensure optimal amplification under universal conditions and lack of interaction during the ligation and PCR steps.

In the implemented strategy, mixtures of multiple PRI-lock probes are ligated on fragmented DNA (Figure 1C). Target recognition is achieved by specific hybridization of both arm sequences, and efficient ligation occurs only if the end nucleotides perfectly match the target [23]. Therefore, the probes confer superior specificity [21,23]. After ligation, the probes are reversibly captured via the desthiobiotin moiety, treated with exonuclease (Figure 1C) and finally individually assayed via real-time PCR on OpenArray™ plates (Figure 1D).

In this study, we characterize the quantification power of circularizable ligation probes over a range of target concentrations and multiple target ratios and report the development of a high-throughput, quantitative multiplex diagnostic assay. The specificity, sensitivity, linear quantification range, and the dynamic detection range of the developed assay were demonstrated using 13 pathogen specific PRI-lock probes, ligated on individual and mixed target DNAs, followed by real-time PCR on OpenArrays™.

## Results

### PRI-lock probe design and evaluation of assay performance

The newly designed probing system was experimentally tested using 13 PRI-lock probes engineered to detect several economically important plant pathogens at different taxonomic levels (Table 1). The target complementary regions were selected as previously described, and the specificity of the probes was verified [21]. Unlike padlock probes, each of the PRI-lock probes was designed with unique primer binding sites allowing quantitative detection. The PRI-lock probes were also engineered with a desthiobiotin moiety between the primers sites for reversible PRI-lock probe capture, washing and release using streptavidin-coated magnetic beads. The introduction of this additional purification step removes excess non-target DNAs and possible enzyme-inhibiting compounds, resulting in increased efficiency of exonuclease treatment and reduced assay background (data not shown).

**Table 1:** Target complementary regions and unique primer sequences for the PRI-lock probes.

Targeted species/group	5' Target complementary sequence (5'-3')	3' Target complementary sequence (5'-3')	Forward primer sequence (5'-3')	Reverse primer sequence (5'-3')
*Phytophthora *spp.	TATCTAGTTAAAAGCA**GA**GACTTTCGTC	CTGCTGAAAGTTGC	TACGAACGTCTTAGCACTCC	GGTGTTGATTCGCGTCTACT
*P. infestans*	TCGATTCGTGGTATGGTTGGCTTCGGCT	CGTTAATGGAGAAATGC	AGAGTCGGTAGGCACTATGG	CGTATGTCGAATGCAGCTGA
*R. solani *AG 2-2	TCTGCCTCACAGGTTCACAGGTGTGTGTGG	TTCCCGTCCATGTC	GAGTTCCCGTGCGTTAGATC	TACGGCGCTTGGGACATGAT
*R. solani *AG 4-1	GGTCCAATAAAGT**T**CCTT**C**CCCCCTAGAAAA	AGTCCAA_**G**GAGAGTA___	CGTGTCCATCGAGCTGCATA	GACGGCATTCAGAGTACGCT
*R. solani *AG 4-2	GACTTCTGTCTACTTAATTCATATAAACTCAATT	CTT_CTACTCCCCCTT_	CTGCCTGTGACTCGTGTATC	AGACCGTATCGTCCACAGTG
*F. oxysporum*	GCGAGTCCCAACACCAAGCTGTGCTTG	GGAACGCGAATTAAC_	CTGGTGCATGTACTCGACTG	ATCAGATCGACTCGGTAGCT
*M. roridum*	CGG**T**GGTGGCCATGCCGTAAAACACC	ACTCGCATTGGAGCT	CATCCAGCTCAACGTATCCA	CCTACTGTGACGCTGTGATG
*V. dahliae*	TTTATACCAACGATACTTCTGAGTGTT	CATCAGTCTCTCTG	ATCTGGATCAACGTCGCGCT	ATACAGTCGTCGGGGTCGAA
*V. alboatrum/V. tricorpus*	TTTATACCAACGATACTTCTGAGTGTTCTTAGTGAAC	GTACATCAGTCTCTTTA	GCATCGGGTTCACGCCTATA	TGAAGCACTGACACGCGAAG
*M. hapla*	GTTTATCGTTGTGAATGGCTGTCGCTGGTG	ATTCGAATAGTCTCAAC	TATGGGTCTTGCTGATACGC	TCCGTCTGTTGAGTTAGGCC
*E. carotovora carotovora*	AAAACCTGTGCGTTCATCGATGCTGAACAT	TCAACGCGAAGGA	AGAATCGTACACGCTGCTGG	AATACGACTGACACGAGCTG
*A. tumefaciens*	TCCGGTTGA**T**AGTTGAGGACA**G**CATTGGAC	GTTGGTCGTCCGCT	CAATACCTGTGACGAGCTGG	ACCCGGTCACTCAGCATATA
*G. Proteo *bacterial spp.	GGCCTTCTTCACACACGCGGCATGGCTGCA	GCTTTACAACCCGAA	ACAGGTCATCGAACTCTCAC	AGAACACGTCAGAGGTCCGT
Internal Ligation Control (ILC)	GGGAGAACACTGCGTGGTTTTCACATAC	GCTTGTGCCTCTCGA	CTATCGCGTGCTAGTCGTCT	ATTCTAATCAATCGTCGCGG

Nucleotides highlighted in bold font, indicate polymorphism within the target group. Nucleotides or gaps owing to deletions used to discriminate from most similar, non-target sequences, are underlined. (No such sequence was found for the PRI-lock probes *G. Proteo *bacterial spp. and *A. tumefaciens*)

In the demonstrated assay, the pathogen detection and quantification are dependent on the ligation and real-time PCR amplification steps. To provide more precise quantification and to correct for the variability arising from the ligation reaction, we designed an Internal Ligation Control (ILC) PRI-lock probe (Table 1) targeting an internal ligation control DNA template. In addition, a PCR control was developed to monitor differences in PCR efficiency.

**Figure 2:**
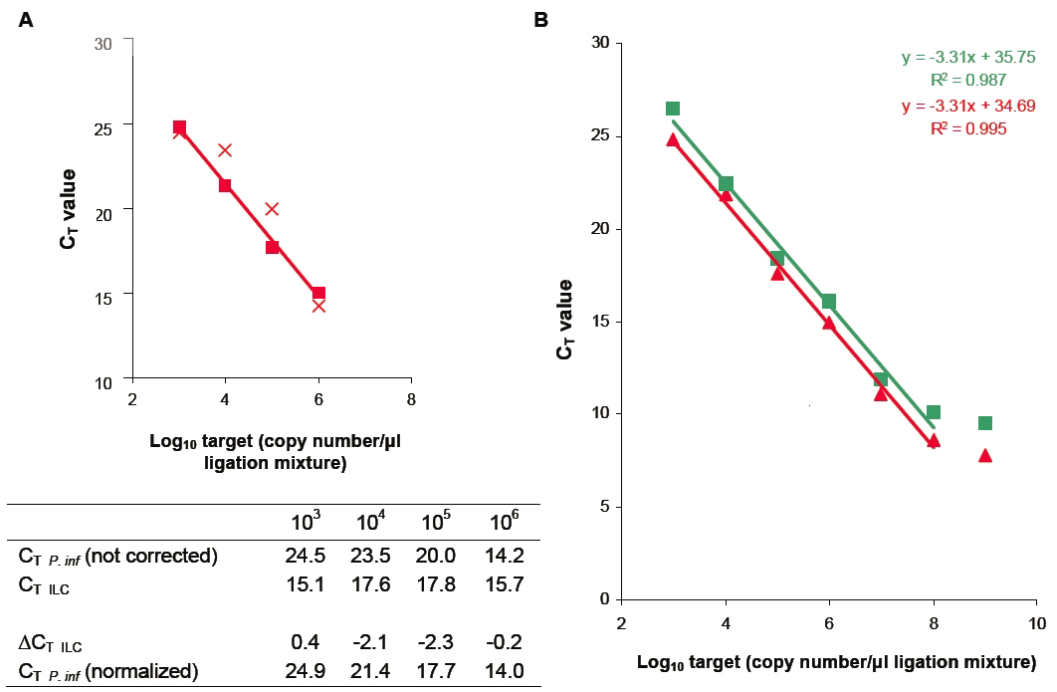
**(A) **Performance of the internal ligation control. The PRI-lock probe mixture was ligated using varying ligation reaction temperatures on a 10-fold serial dilution of *P. infestans *target and amplified in conventional real-time PCR using the *P. infestans *and the ILC PRI-lock probe specific primer pairs. The *P. infestans *PRI-lock probe C_T _value was normalized using the ILC C_T _value as described in the Materials and Methods section. *P. infestans *C_T _values without normalization (x). Normalized *P. infestans *C_T _values (▪). **(B) **Calibration curves to assess the sensitivity and linear range of quantification of the *P. infestans *PRI-lock probe in conventional real-time PCR (red) and in the Biotrove OpenArray™ platform (green). The PRI-lock probe mixture was ligated on a 10-fold *P. infestans *target dilution series and amplified in real-time PCR using the *P. infestans *PRI-lock probe specific primer pair. C_T _values were normalized using the ILC control PRI-lock probe. Data represent average C_T _values of six PCR replicates (n = 6). The standard deviation error bars fall within the data squares.

The developed PRI-lock probe system was validated in several steps. To evaluate the specificity of the designed PRI-lock probes, a mixture of 14 probes (13 target probes plus one internal ligation control) was ligated on various individual DNA targets. The samples were screened for amplification with all the individual PRI-lock probe specific primer pairs in conventional real-time PCR. All the target sequences were specifically detected without exception (Table 2) and no amplification of ligated PRI-lock probes with other, non-cognate primer pairs occurred (data not shown). In this and all subsequent experiments, the amplification efficiency of the PCR control was found to be uniform (data not shown) and the C_T _values were normalized using the ILC as shown in figure 2A. To further ensure specificity towards the targeted pathogens, 3 non-target organisms with very similar ligation sites (1, 3 and 7 mismatches compared to the perfect ligation sequence) for the PRI-lock mixture were tested. The rest of the probes discriminated their targets from the most similar non-target sequence based on more than 7 mismatches and were not tested. In agreement with previous results [21], no signal was observed in the presence of the non-target organisms with very similar ligation sites (Table 3), demonstrating the specific target recognition by the designed PRI-lock probes.

**Table 2:** Specificity and multiplexing of the PRI-lock probe system in conventional real-time PCR and in the Biotrove OpenArray™ for single and multiple targets.

	Single target		Multiplex mix 1		Multiplex mix 2		Multiplex mix 3	
PRI-lock Probe	C_T_(AB)	C_T_(BT)	C_T_(AB)	C_T_(BT)	C_T_(AB)	C_T_(BT)	C_T_(AB)	C_T_(BT)
*Phytophthora *spp.	15.5 (0.14)	16.4 (0.17)	15.7 (0.10)	16.7 (0.25)	16.1 (0.04)	16.8 (0.12)	16.3 (0.16)	15.9 (0.09)
*P. infestans*	14.8 (0.12)	15.4 (0.18)	--	--	15.2 (0.08)	15.7 (0.12)	15.1 (0.02)	15.0 (0.08)
*A. tumefaciens*	16.4 (0.24)	16.2 (0.04)	--	--	--	--	15.8 (0.28)	15.9 (0.06)
*G. Proteo *bacterial spp	17.0 (0.11)	17.7 (0.08)	--	--	--	--	17.7 (1.03)	17.1 (0.04)
*M. roridum*	16.2 (0.07)	--	16.4 (0.02)	17.2 (0.05)	--	--	16.7 (0.12)	17.0 (0.16)
*F. oxysporum*	17.4 (0.11)	--	--	--	--	--	17.2 (0.08)	17.3 (0.04)
*E. carotovora carotovora*	15.1 (0.15)	--	--	--	--	--	15.2 (0.24)	15.4 (0.05)
*V. dahliae*	16.9 (0.01)	--	--	--	--	--	16.1 (0.04)	15.8 (0.03)
*V. alboatrum/V. tricorpus*	19.6 (0.11)	--	--	--	--	--	19.9 (0.29)	18.0 (0.10)
*M. hapla*	17.0 (0.30)	--	--	--	--	--	18.2 (0.23)	16.9 (0.11)
*R. solani *AG 2-2	16.3 (0.03)	--	--	--	15.8 (0.10)	17.1 (0.12)	16.2 (0.03)	16.2 (0.04)
*R. solani *AG 4-1	14.0 (0.02)	--	--	--	14.6 (0.13)	15.0 (0.11)	14.5 (0.13)	14.4 (0.09)
*R. solani *AG 4-2	19.3 (0.10)	--	--	--	19.5 (0.14)	20.0 (0.18)	19.3 (0.07)	18.8 (0.03)

The PRI-lock probes were ligated on 10^6 ^targets/μl ligation mixture. C_T _values were normalized using the ILC control PRI-lock probe. Data represent average C_T _values of three PCR replicates in the conventional real-time platform (n = 3) and of four PCR replicates in the Biotrove OpenArray™ (n = 4). Standard deviations are indicated between brackets. AB: samples run on the conventional real-time PCR platform. BT: Samples tested on the Biotrove OpenArray™ platform. Multiplex mix 1: *P. soj*. and *M. ror*. DNA. Multiplex mix 2: *P. inf*., *R. sol*. AG 2-2, *R. sol*. AG 4-1 and *R. sol*. AG 4-2 DNA. Multiplex mix 3: ligation mixture containing all the DNA targets indicated in table 2. --: not tested.

**Table 3:** Specificity of the PRI-lock probe system in conventional real-time PCR and in the Biotrove OpenArray™ for non-target organisms with closely related ligation sites.

	*Pythium undulatum *cnt *Phytophthora *spp.(1)		*Phytophthora sojae *cnt *P. infestans *(3)		*Myrothecium verrucaria *cnt *M. roridum *(5)	
PRI-lock Probe	C_T_(AB)	C_T_(BT)	C_T_(AB)	C_T_(BT)	C_T_(AB)	C_T_(BT)
*Phytophthora *spp.	nd	nd	15.5 (0.11)	16.1 (0.10)	nd	nd
*P. infestans*	nd	nd	nd	nd	nd	nd
*M. roridum*	nd	nd	nd	nd	nd	nd

The PRI-lock probes were ligated on 10^6 ^targets/μl ligation mixture. C_T _values were normalized using the ILC control PRI-lock probe. Data represent average C_T _values of three PCR replicates in the conventional real-time platform (n = 3) and of four PCR replicates in the Biotrove OpenArray™ (n = 4). Standard deviations are indicated between brackets. AB: samples run on the conventional real-time PCR platform. BT: Samples tested on the Biotrove OpenArray™ platform. Number of discriminating nucleotides in the ligation region are indicated between brackets. nd: not detectable. cnt: closest non target.

To evaluate the performance of the PRI-lock system in a multiplex setting, the probe mix was ligated on several target DNA mixtures and analyzed in conventional real-time PCR. Multiple target DNA sequences were detected with no statistically significant change in the observed C_T _values compared with the C_T _values observed for single-target detection (Table 2).

In diagnostic applications, we expect to quantify several target organisms over large concentration differences. Thus, to investigate the sensitivity and linear quantification range of the PRI-lock probe system in conventional real-time PCR, a 10-fold dilution series of a *Phytophthora infestans *target was detected and quantified. As shown in figure 2B, a linear quantification range of 6 orders of magnitude was achieved with a sensitivity of 10^3 ^*P. infestans *target copies/μl initial ligation mixture. Moreover, the correlation between the logarithmic target concentration in the ligation mixture and the observed C_T _value was very high (R^2 ^= 0.995). All the other PRI-lock probes showed similar results (data not shown), demonstrating that the PRI-lock system can be used for reliable quantification over a wide concentration range.

### Application of the PRI-lock based multiplex quantitative detection on the Biotrove OpenArray™ platform

On a conventional real-time PCR machine, analysis of a single sample however, would typically require 14 separate reactions, one for each probe, making large scale screenings time consuming, laborious and expensive. A solution was offered by the Biotrove OpenArray™ system, which provides a platform for the parallel analysis of 48 samples against the 14 PRI-lock probes. To compare the performance of the PRI-lock system in the Biotrove OpenArray™ with the performance in conventional real-time PCR, samples containing single or multiple targets were ligated and analyzed on the Biotrove OpenArray™ platform. The obtained C_T _values were found to be similar to the C_T _values observed in the conventional real-time PCR (Table 2), although the number of target copies per PCR was lower in the OpenArray™. Similarly to the standard real-time PCR platform, a linear quantification range of 6 orders of magnitude was achieved with a sensitivity of 10^3 ^*P. infestans *target copies/μl initial ligation mixture (Figure 2B).

**Figure 3:**
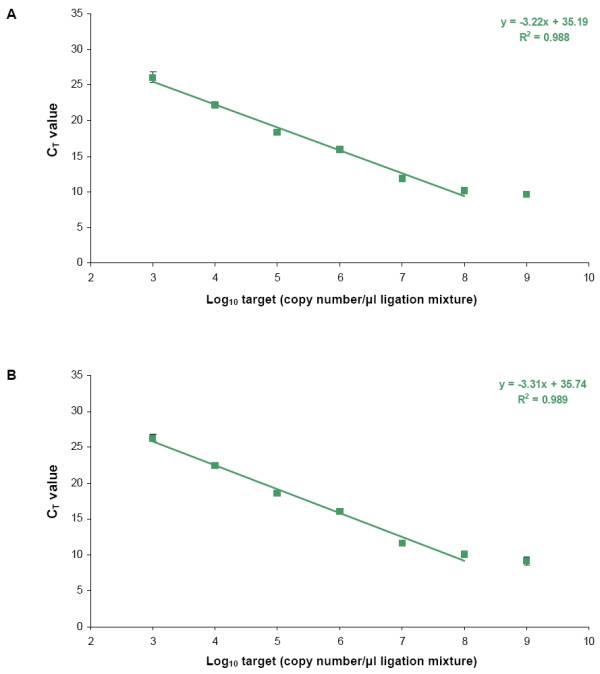
Calibration curves to assess the reproducibility of the Biotrove OpenArray™ platform and the PRI-lock system. (**A**) Inter-array variation: The PRI-lock probe mixture was ligated on a 10-fold serial dilution of *P. infestans *target and amplified on the Biotrove OpenArray™ platform. Samples were tested on three different OpenArrays™ (n = 3). Data represent averages of six PCR replicates (n = 6). The error bars represent the standard deviations. (**B**) Assay-to-assay reproducibility: Three separate whole assay repeats ligation reactions were performed on 10-fold serial dilution of *P. infestans *target and amplified in the Biotrove OpenArray™ platform. Data represent averages of 3 separate ligation experiments (n = 3) with each, four PCR replicates (n = 4). C_T _values were normalized using the ILC PRI-lock probe. The error bars represent the standard deviations (Sometimes the standard deviation error bars fall within the data squares).

To test the reliability of the developed assay, we analyzed the inter-array variation of the OpenArray™ platform and the assay-to-assay reproducibility of the PRI-lock probe based quantification. To test the inter-array PCR variation, a PRI-lock probe mixture ligated on a 10-fold dilution series of *P. infestans *target was assayed in triplicate on three separate arrays (Figure 3A). The standard deviations of the data points ranged from 0.05 to 0.71 C_T _values, indicating a low inter-array variation. Next, a dilution series of *P. infestans *target DNAs was quantified by performing each ligation reaction in triplicate. (Figure 3B). The observed assay-to-assay variability was low, with standard deviations ranging from 0.18 to 0.58 C_T _values. Assay-to-assay reproducibility tests using the other PRI-lock probes showed similar results (data not shown), demonstrating the high reproducibility and quantitative reliability of the PRI-lock probe detection system across multiple assays.

### Quantification and validation

To enable accurate pathogen quantification of unknown samples, calibration curves were constructed for each of the 13 PRI-lock probe targets. Because the ligation of different probes is independent, the calibration curves could be constructed based on C_T _values measured in a multiplex setting. The PRI-lock probes were ligated on a 10-fold dilution series of all target DNA sequences and amplified in the Biotrove OpenArray™ platform. The obtained calibration curves showed that all the target pathogens were quantified over at least 5 orders of magnitude (Table 4). The correlation between the logarithmic target concentration and the observed C_T _value was very high for all the detected pathogens (lowest R^2 ^= 0.984, average R^2 ^= 0.993).

**Table 4:** Quantification and sensitivity of the developed assay on the Biotrove OpenArray™ platform.

PRI-lock probe	Calibration curve formula	R^2 ^value	Detection limit (copy number/μl ligation mixture)	Linear quantification range (copy number/μl ligation mixture)
*Phytophthora *spp.	y = -3.61x + 38.1	0.995	10^4^	10^8 ^– 10^4^
*P. infestans*	y = -3.31x + 35.7	0.989	10^3^	10^8 ^– 10^3^
*R. solani *AG 2-2	y = -3.49x + 37.9	0.994	10^4^	10^8 ^– 10^4^
*R. solani *AG 4-1	y = -3.49x + 35.8	0.994	10^3^	10^9 ^– 10^3^
*R. solani *AG 4-2	y = -3.35x + 39.2	0.998	10^3^	10^8 ^– 10^4^
*F. oxysporum*	y = -3.47x + 39.1	0.990	10^4^	10^8 ^– 10^4^
*M. roridum*	y = -3.15x + 36.6	0.984	10^4^	10^9 ^– 10^4^
*V. dahliae*	y = -3.42x + 36.3	0.996	10^3^	10^8 ^– 10^3^
*V. alboatrum/V. tricorpus*	y = -3.40x + 38.8	0.991	10^3^	10^8 ^– 10^4^
*M. hapla*	y = -3.39x + 38.1	0.989	10^4^	10^8 ^– 10^4^
*E. carotovora carotovora*	y = -3.51x + 36.3	0.997	10^3^	10^9 ^– 10^3^
*A. tumefaciens*	y = -3.36x + 36.5	0.995	10^3^	10^8 ^– 10^3^
*G. Proteo *bacterial spp	y = -3.51x + 38.1	0.995	10^4^	10^8 ^– 10^4^

(x) = Log_10_(copy number target input/μl ligation mixture). (y) = C_T _value.

The corresponding calibration equations enable us to determine the absolute number of targets in the analyzed samples. To demonstrate this, we tested samples of known amounts of DNA targets in different ratios as well as genomic DNAs in different concentration ratios. First, known amounts of *Phytophthora sojae *and *Erwinia carotovora carotovora *DNA targets were mixed in reciprocal ratios of 1:10^3 ^and 1:10^4 ^(Table 5). The calculated target numbers/μl ligation mixture based on the observed C_T _values in combination with calibration formulas, were very similar to the original target inputs (Table 5). Second, the assay was evaluated using artificial mixtures of different ratios of genomic DNA concentrations. Using the calibration formulas of the corresponding PRI-lock probes and the observed C_T _values, the original target number for each detected target was calculated (Table 6). In the first example, an input of 100 pg *Fusarium oxysporum *gDNA/μl ligation mixture resulted in a C_T _value for the *F. oxysporum *PRI-lock probe of 17.2. With the corresponding calibration formula for the *F. oxysporum *PRI-lock probe, we calculated that in the original ligation sample 10^6.3 ^copies *F. oxysporum *targets/μl ligation mixture were present (Table 6). In the second example, 100 times less *F. oxysporum *DNA was added compared to the first sample. In this case, the observed C_T _value of 24.5 yielded a calculated copy number of 10^4.2 ^*F. oxysporum *targets/μl ligation mixture, closely matching the actual 100 times difference in target input (Table 6).

**Table 5:** PRI-lock assay validation. Analysis of target DNAs mixed in different ratios.

Template (Log_10 _target (copy number/μl ligation reaction))		Ratio	Observed C_T _values (SD) in PRI-lock specific PCR		Observed pathogen template (SD) (Log_10 _target (copy number/μl ligation reaction))	
			
*P. sojae*	*E. carotovora carotovora*		*Phytophthora *spp.	*E. carotovora carotovora*	*Phytophthora *spp.	*E. carotovora carotovora*
8	5	1000: 1	9.4 (0.30)	19.3 (0.23)	7.9 (0.08)	4.9 (0.07)
8	4	10000: 1	9.3 (0.06)	22.7 (0.34)	8.0 (0.02)	3.9 (0.10)
5	8	1: 1000	20.6 (0.20)	8.8 (0.14)	4.8 (0.06)	7.9 (0.04)
4	8	1: 10000	24.2 (0.80)	8.9 (0.07)	3.8 (0.22)	7.8 (0.02)

Target numbers were calculated using the C_T _values and the corresponding calibration curves. Data represent 2 ligation replicates (n = 2), each with 4 qPCR replicates (n = 4).

**Table 6:** PRI-lock assay validation. Analysis of genomic DNA targets mixed in different ratios.

Genomic DNA template (pg/μl ligation mixture)						Log_10 _target (copy number/μl ligation mixture) (SD)						
	
*F. oxysporum*	*P. fragariae*	*M. roridum*	*R. solani *AG 4-1	*R. solani *AG 4-2	*E. c. c*.	*F. oxysporum*	*P. fragariae*	*M. roridum*	*R. solani *AG 4-1	*R. solani *AG 4-2	*E. c. c*.	*Gamma Proteo *bacterial spp.
	
100	100	100	--	--	10	6.3 (0.10)	6.8 (0.08)	6.8 (0.07)	--	--	3.5 (0.09)	4.6 (0.10)
1	0.1	100	--	--	--	4.2 (0.12)	3.6 (0.27)	6.7 (0.05)	--	--	--	--
100	0.01	0.01	--	--	100	6.1 (0.14)	nd	nd	--	--	4.4 (0.08)	5.5 (0.13)
10	100	1	--	--	1	5.1 (0.08)	6.8 (0.09)	4.4 (0.11)	--	--	**2.8 (0.17)**	3.5 (0.11)
10	0.01	1	--	--	10	5.2 (0.17)	nd	4.5 (0.04)	--	--	3.5 (0.20)	4.5 (0.16)
10	0.001	1	--	--	0.01	5.1 (0.09)	nd	4.4 (0.20)	--	--	nd	nd
10	1	0.1	--	--	100	5.1 (0.15)	4.6 (0.14)	3.3 (0.04)	--	--	4.4 (0.07)	5.4 (0.05)
10	10	10	10	10	10	5.0 (0.07)	5.6 (0.05)	5.4 (0.06)	4.3 (0.08)	5.0 (0.20)	3.2 (0.23)	4.3 (0.08)
--	--	--	100	1	--	--	--	--	5.7 (0.06)	4.0 (0.23)	--	--
0.1	--	--	1	--	10	**3.8 (0.20)**	--	--	3.3 (0.16)	--	3.5 (0.10)	4.6 (0.10)

Target numbers were calculated using the C_T _values and the corresponding calibration curves. Data highlighted in bold font, indicate data outside the linear range. Data represent 2 ligation replicates (n = 2), each with 4 qPCR replicates (n = 4). Nd: not detectable. *E.c.c*.:*Erwinia carotovora carotovora*

## Discussion

In this study, we demonstrated a specific, multiplex, PRI-lock probe-based, high-throughput detection assay using the Biotrove OpenArray™ platform for the detection and quantification of plant pathogens. The described application serves as a model for the development of rapid, molecular detection systems that offer an unprecedented combination of specificity, high throughput capabilities and robust target quantification.

To evaluate the designed PRI-lock probes and corresponding primer pairs, the probes were ligated on single target sequences and tested using conventional real-time PCR. In all cases, the targets were correctly detected and no false positives were observed, indicating highly specific ligation of the PRI-lock probes on their respective targets. Furthermore, a ligation reaction performed on non-target organisms with very similar ligation target sites did not result in detectable fluorescent signals. In agreement with the results obtained previously [21], we demonstrated that the PRI-lock probe system could discriminate non-target sequences on a single nucleotide difference. This is of prime importance, because non-target organisms with very similar ligation target sites might be present in diagnostic samples. In addition, the influence of multiple targets on PRI-lock probe based detection was tested, and it was demonstrated that the presence of multiple targets had no statistically significant influence. Our multiplex detection system provided truly independent detection of the different pathogens, with no evidence of inhibition due to possible ligation competition.

Quantitative diagnostic assays require a linear range of quantification of several orders of magnitude. Ligation of short oligonucleotides was previously used successfully for the characterization of gene expression and gene copy number [24,25]. In these cases, however, the potential target concentration range was much lower. We showed that ligation of PRI-lock probes can reflect well the target quantity over at least 5 orders of magnitude.

The sensitivity of detection was determined by testing a 10-fold dilution series of ligation targets for all the PRI-lock probes. Sensitivities of 10^3 ^and 10^4 ^target copies/μl initial ligation mixture were achieved, depending on the PRI-lock probe. Assuming *e.g*. an average rRNA gene copy number of 200 times per fungal or oomycete genome, our assay would require between 50 and 500 target genomes per 10 μl initial ligation mixture for reliable detection. For organisms with lower rRNA copy numbers, such as bacteria, the assay would require more target genomes input compared to the previous example. However, the final input in the nanoliter real-time reactions is only a fraction of the original ligation mixture (< 0.1%). To increase the final assay detection sensitivity, not the ligation sensitivity but the copy number input of ligated PRI-locks per nanoliter reaction should be increased. Compared with other circularization probe-based assays, we already increased the applied PRI-lock probe concentration in our assay [21,26,27]. Increasing the PRI-lock probe concentration in the ligation mixture even further does not seem to yield further gains in sensitivity (not shown). A pre-amplification of the ligated PRI-lock probes therefore, could serve as an alternative strategy to increase the final assay sensitivity. PCR-based amplification however, often leads to amplification biases, changing the ratios between targets in the original biological sample [28]. Non-PCR-based strategies, like linear amplification, have been shown to minimize amplification biases and generally conserve the target ratios originally present in the template mixture [29,30]. Incorporating a strategy with a linear amplification of the ligated probes into the PRI-lock based detection assay, therefore, seems to represent a potential way of increasing detection sensitivity, without compromising quantitative power.

The target copy number used as template in the OpenArray™ is lower than the copy number in the conventional real-time PCR. Despite the lower copy number input, the observed C_T _values and the linear detection range of the developed PRI-lock system were about the same for the two real-time platforms (Figure 2B). It is known that, in nanoliter reactions, the concentration of the amplified products reaches the threshold concentration needed to provide a significant fluorescent signal sooner than in microliter reactions [31]. This property was previously exploited to detect as little as one template molecule in nanoliter PCR amplifications, without the need to increase the PCR assay cycle number [19,31]. Together with the improved C_T _calling algorithm of the Biotrove OpenArray™ platform [19], this results in a performance equal to conventional real-time PCR despite the reduction in template target copy number.

The robustness of the PRI-lock system was tested by analyzing the inter-array variation of the OpenArray™ platform and the assay-to-assay reproducibility of the PRI-lock probes. Both, the inter-array and assay-to-assay variation were very low, demonstrating the reproducibility and quantitative reliability of the PRI-lock detection assay. The developed quantitative multiplex detection assay was validated by testing various artificial mixtures of target DNAs. We demonstrated that the calculated number of *P. infestans *and *E. carotovora carotovora *input targets, based on the observed C_T _values and the corresponding calibration formulas, were highly similar to the actual *P. infestans *and *E. carotovora carotovora *input target numbers. In a further test, *P. infestans *and *E. carotovora carotovora *targets were mixed in different ratios, using target concentrations within the linear detection range of the entire probe set. For both targets, a reciprocal dynamic range of 10^4 ^was established in a background of 20 ng non-target DNA. The observed dynamic range is an improvement over most previously developed multiplex pathogen detection assays, where the dynamic range was often limited to 100–1000 [21,32]. In the Biotrove OpenArray™ platform, target PCR amplifications are completely independent of each other, and, consequently, no PCR competition among the different ligated PRI-lock occurs. The dynamic range of detection in the PRI-lock detection assay is therefore, as long as the PRI-lock probes ligate on their respective targets, practically unlimited. Finally, artificial mixtures of genomic DNAs in different concentration ratios were tested. The components of the pathogen mixture were identified, and the original target input was calculated using the calibration formulas. Moreover, the ratios of the targets among the different ligation samples were correctly identified as well. The consistent, 10-fold discrepancy in observed target amounts between the *E. carotovora carotovora *and *G. Proteo *bacterial PRI-lock probes is a consequence of the different ligation regions for each probe within the genomic DNA of *E. carotovora carotovora*. Apparently, the copy number of the 16S rRNA gene detected by the *G. Proteo *bacterial PRI-lock is approximately 10-fold higher than the *recA *gene detected by the *E. carotovora carotovora *PRI-lock probe.

Circularization probes have previously been applied successfully for the detection of multiple plant pathogens in diagnostic samples [21], but without the ability to quantify target numbers. To our knowledge, this report presents the first time that numerous plant pathogens could be simultaneously and accurately quantified using specific circularization probes in a single assay. For future applications, however, higher multiplexing is intended and therefore, the number of PRI-lock probes will be increased. Currently, assay background is considered as the biggest obstacle for increasing the level of multiplexing in traditional circularization probe-based diagnostic assays. Traditional circularization probes contain generic primer sites for PCR amplification [21]. Multiplex PCR via general primer sites carries the potential for competition during amplification, with a cumulative increase in background, which reduces assay sensitivity. In contrast, each PRI-lock probe carries a unique pair of primer binding sites, unrelated to the sequences of all the other probes. Circularized PRI-lock probes can therefore be amplified individually. Increasing the level of multiplexing would not be expected to increase the background signal. To further guarantee low background, even in highly multiplex settings, we are currently developing a universal TaqMan^® ^probe which hybridizes to the generic sequence incorporated in all the PRI-lock probes. In addition, including a universal TaqMan^® ^probe should speed up data analysis, and therefore sample throughput, since there is no need to conduct amplicon dissociation curve analysis. Given the independent PCR amplification of the ligated PRI-lock probes and the three slide capacity of the OpenArray™ NT Cycler, it should be feasible to engineer ultra-high throughput arrays for the quantitative detection of hundreds of targets simultaneously.

## Conclusion

To date, most multiplex pathogen detection has been performed using traditional hybridization microarrays [8,33]. Although such platforms typically allow highly multiplex detection, they generally offer relatively low sample throughput and yield limited quantitative information compared to qPCR [34]. In this study, we described a high-throughput diagnostic system that combines very specific multiplex pathogen detection with accurate quantification over a range of target concentrations. The PRI-lock probes, combined with the OpenArray™ system, offer a flexible and adaptable design of high-throughput, quantitative multiplex detection assays, since the target recognition is separated from further downstream processing. It should be noted, although we have demonstrated that large quantities of non-target DNA do not influence the accuracy of PRI-lock probe-based detection, PRI-lock performance remains to be examined within field applications. The PRI-lock system described is readily modifiable and expandable to include an almost unlimited range of potential targets, providing an easily accessible platform for versatile diagnostic applications.

## Methods

### Nucleic acids used in the study

Microorganisms were derived from the culture collection of Plant Research International BV (Table 7). Genomic DNAs from all microorganisms were isolated using the Puregene Genomic DNA isolation kit (Gentra/Biozym, Landgraaf, the Netherlands) according to the manufacturer's instructions. Ligation targets for assay optimization were generated using 500 pg extracted genomic DNA as PCR template. The Internal Transcribed Spacer (ITS) regions of the fungal and oomycetal rRNA operons were amplified using the primers ITS1 and ITS4 [35] and the ITS region of *Meloidogyne hapla *spp. was amplified using the primers F194 and F195 [36]. Sequences of the primers for partial amplification of the bacterial 16S rRNA genes were derived from Rochelle *et. al*. [37] and the *Erwinia carotovora carotovora recA *gene was amplified using general *recA *primers [38]. Primers (forward 5'-CTAATTTTCGGTCCAACT-3', reverse 5'-GCTTAACTCTGGCCTTG-3') for partial amplification of the *Agrobacterium tumefaciens ipt *gene were designed using Oligo 6.0 software (Molecular Biology Insights, Inc., Cascade, USA). For each PCR, an initial 10 min incubation at 95°C was followed by 40 cycles consisting of a 30 s incubation at 95°C, an annealing step at 60°C for 30 s (ITS), 55°C for 60 s (16S), 47°C for 60 s (*recA*), or 50°C for 30 s (*ipt*), and an elongation step at 72°C for 60 s. After the last cycle, the reaction was immediately cooled to 4°C. The PCR amplicons of the different microorganisms were purified using the QIAquick^® ^PCR Purification Kit (QIAGEN GmbH, Hilden, Germany) and quantified by comparison to a DNA ladder after gel electrophoresis. Subsequently, the number of copies per μl PCR product of each amplicon was calculated.

**Table 7:** Isolates of plant pathogenic species and subgroups used in this study.

Phylum	Order	Species	Isolate
Oomycota	Peronosporales	*Phytophthora infestans*	VK98014
		*Phytophthora fragariae*	FVF01
		*Phytophthora sojae*	F. Govers 6497
		*Pythium undulatum*	CBS 157.64
Basidiomycota	Ceratobasidales	*Rhizoctonia solani *AG 2-2	IIIB 02–337 IRS
		*Rhizoctonia solani *AG 4-1	PRI 4R91
		*Rhizoctonia solani *AG 4-2	PRI 4R22
Ascomycota	Hypocreales	*Fusarium oxysporum *f. sp. *radicis-lycopersici*	364N2
		*Myrothecium roridum*	PRI-15.2
		*Myrothecium verrucaria*	CBS 189.46
	Phyllacorale	*Verticillium dahliae*	809.97
		*Verticillium alboatrum*	40.1
Nematoda	Tylenchida	*Meloidogyne hapla*	HBA
Proteobacteria	Enterobacteriales	*Erwinia carotovora *sp.*carotovora*	103
	Rhizobiales	*Agrobacterium tumefaciens*	PRI-IS4

A PCR control was developed to monitor differences in PCR efficiency within and between different 96-wells plates and different OpenArrays™. To this end, a 99 bp PCR control template (5'-CTAACGAATCTGGGACGTGCATCCGGTCTCATCGCTGAATCGCTCGTGAGGGCAGGGCCGGGAGGGGGGTCCGCAGGCGCAACACTGTAGTCGGTGCTA-3') was amplified using the forward 5'-CTAACGAATCTGGGACGTGC-3' and reverse 5'-TAGCACCGACTACAGTGTTG-3' primer pair.

The PRI-lock probes listed in table 1 and all the other oligonucleotides used in this study, were synthesized by Eurogentec SA (Seraing, Belgium)

### PRI-lock probe design

The PRI-lock probe target complementary regions were engineered according to previously described design criteria [21] and were connected by a 60 bp compound linker sequence. The linker sequence contained a 20 bp, generic sequence and two, unique primer binding sites for specific PCR amplification (Table 1). All primer pairs have equal melting temperatures to allow universal SYBR-Green based detection in real-time PCR. The primer pairs were chosen from GeneFlex™ TagArray set (Affymetrix) so as to minimize PRI-lock probe secondary structures and optimize both, primer *T*_*M *_and primer specificity. Potential for secondary structures, primer *T*_*M *_and primer specificity were predicted using Visual OMP 5.0 software (DNA Software Inc., Michigan, USA). The prediction parameters were set to match ligation ([monovalent] = 0.025 M; [Mg^2+^] = 0.01 M; T = 65°C, [probe] = 250 pM) and PCR conditions ([monovalent] = 0.075 M; [Mg^2+^] = 0.005 M, T = 60°C). When necessary, PRI-lock probe arm sequences were adjusted to avoid strong secondary structures that might interfere with efficient ligation as described previously [21].

Between the primer sites we introduced a thymine-linked desthiobiotin molecule for specific capturing and release with streptavidin-coated magnetic beads. The rationale of using desthiobiotin instead of biotin is the approximately 1000 times lower affinity for streptavidin [22,39], which permits the reversible release of the PRI-lock probes (see below).

### Ligation

PCR fragments and genomic DNAs were used as templates for ligation in the indicated amount. Prior to ligation, genomic DNA was fragmented by digestion using EcoRI, HindIII and BamHI (New England Biolabs Inc., Ipswich, USA) for 15 min at 37°C. Cycled ligation was performed in 10 μl reaction mixture containing 20 mM Tris-HCl, pH 9.0, 25 mM KCH_3_COO, 10 mM Mg(CH_3_COO)_2_, 1 mM NAD, 10 mM DTT, 0.1 % Triton X-100, 20 ng sonicated salm sperm DNA and 20 U Taq ligase (New England Biolabs Inc., Ipswich, USA). Titration experiments with different concentrations of individual PRI-lock probes were performed to achieve comparable PRI-lock performance in terms of C_T _values and linear quantification range. For multiplex detection, the optimized concentrations of the individual PRI-lock probes ranged from 250 pM to 2.5 nM. Reaction mixtures were prepared on ice and rapidly transferred into a thermal cycler. Before ligation, samples were denatured at 95°C for 5 min. The samples were subsequently subjected to 20 cycles of 30 s at 95°C and 5 min at 65°C, followed by enzyme inactivation at 95°C for 15 min.

To monitor the ligation efficiency and provide a reference for normalization, an Internal Ligation Control (ILC) PRI-lock probe was constructed. The target complementary regions of the ILC PRI-lock detect an artificial DNA sequence, of which a fixed amount was added to each ligation reaction, resulting in a standard ILC C_T _value of 15.5 for the AB7500 and the Biotrove OpenArray™. Variation in the ligation reaction was monitored by comparing the observed ILC C_T _values with the fixed ILC C_T _value. The C_T _values were standardized using the following equation:

C_T_(x)_AB7500/Biotrove _= (15.5 - C) + C_T_o(x)

C_T_(x): the standardized C_T _value for PRI-lock probe (x)

C: observed ILC C_T _value

C_T_o(x): observed C_T _value for PRI-lock probe (x)

AB7500: 7500 Real-Time PCR system, Applied Biosystems

Biotrove: OpenArray™ NT Cycler, Biotrove Inc.

### Probe capture, elution and exonuclease treatment

After ligation, 30 μl distilled water was added to each reaction and the samples were denatured at 95°C for 10 minutes and rapidly transferred onto ice afterwards. Capturing of the desthiobiotin moiety of the PRI-lock probes was performed in 80 μl capturing mixture containing 1 M NaCl, 5 mM Tris-HCl, pH 7.5, 0.5 mM EDTA, 0.1 M NaOH and 200 μg magnetic MyOne™ Streptavidin C1 Dynabeads^® ^(Dynal Biotech ASA, Oslo, Norway) and rotated at 4°C for 1 hour. Subsequently, samples were centrifuged at 2000 × g for 10 s, the Dynabeads^® ^were collected and separated from the sample via application of a magnetic field and the Dynabeads^® ^were washed with 100 μl washing solution containing 100 mM Tris-HCl, pH 7.5 and 50 mM NaCl. Consequently, the non target DNA and possible co-extracted enzyme inhibitors are washed away. The Dynabeads^® ^were re-suspended in 10 μl distilled water and incubated at 95°C for 10 minutes, allowing quantitative elution of the PRI-lock probes from the Dynabeads^® ^[40]. Samples were rapidly transferred onto ice afterwards, and the empty magnetic streptavidin beads were removed via application of a magnetic field, leaving the washed PRI-lock probes in the solution. Next, 10 μl of exonuclease mixture [10 mM Tris-HCl, pH 9.0, 4.4 mM MgCl_2_, 0.1 mg/ml BSA, 0.5 U Exonuclease I (USB Europe GmbH, Staufen, Germany) and 0.5 U Exonuclease III (USB Europe GmbH, Staufen, Germany)] was added to each reaction, and the samples were incubated at 37°C for 30 min, followed by enzyme inactivation at 95°C for 2.5 h.

### Real-time PCR

Amplification of ligated PRI-lock probes was monitored in real-time using the 7500 Real-Time PCR system (Applied Biosystems, Foster City, USA). Reactions were performed in 1× SYBR^® ^Green PCR Master Mix (Applied Biosystems, Foster City, USA) containing SYBR green I dye, AmpliTaq Gold^® ^DNA Polymerase, dNTPs with dUTP, passive Reference 1, optimized buffer components, 0.12 μl AmpErase^® ^Uracil N-glycosylase (Applied Biosystems, Foster City, USA), 0.77 μM of each primer, 0.5 pg PCR control template and 2.5 μl ligated PRI-lock mixture as template. The reaction mixture was initially incubated at 50°C for 2 min, followed by 10 min denaturation at 95°C, and 33 cycles of 15 s at 95°C and 1 min at 60°C. Based on the calibration curves, the limit of the linear range of quantification was around cycle 27 and therefore, the maximum number of PCR cycles was set to 33. Fluorescence was recorded in the 60°C step of each cycle, and finally, the amplicon specificity was determined by studying the individual melting curves.

### Biotrove OpenArray™ real-time PCR

Amplification of ligated PRI-lock probes was followed in real-time using an OpenArray™ NT Cycler (BioTrove Inc., Woburn, USA). OpenArray™ subarrays were pre-loaded by Biotrove with the selected primer pairs. Each primer pair was spotted in duplicate. Primer dilution experiments were performed to optimize primer concentrations in the OpenArray™ slides. Concentrations were optimized to obtain minimum primer carry over during sample loading and maximum C_T _values after PCR amplification. The final assay concentration for all the spotted primer pairs was 128 nM except the *V. dahliae *primer pair, which was 64 nM.

Samples were loaded into OpenArray™ plates using the OpenArray™ NT Autoloader according to the manufacturer's protocols. Each subarray was loaded with 5.0 μl mastermix containing 2.5 μl ligated PRI-lock mixture and reagents in a final concentration of 1× LightCycler^® ^FastStart DNA Master SYBR Green I mix (Roche Diagnostics GmbH, Mannheim, Germany), 0.2% Pluronic F-68 (Gibco, Carlsbad, USA), 1 mg/ml BSA (Sigma-Aldrich, St Louis, USA), 1:4000 SYBR Green I (Sigma-Aldrich), 0.5% (v/v) Glycerol (Sigma-Aldrich), 8% (v/v) deionzed formamide (Sigma-Aldrich) and 1.0 pg PCR control template.

The PCR OpenArray™ thermal cycling protocol consisted of 90°C for 10 min, followed by cycles of 28 s at 95°C, 1 min at 55°C and 70 s at 72°C (imaging step). The maximum number of PCR cycles was set to 27. Due to the smaller reaction volume in the OpenArray™ plates, additives in the PCR master mix and different surface properties, the annealing temperature of the Biotrove OpenArray™ system had to be adjusted to mimic the actual PCR conditions in the 7500 Real-Time PCR system. Simulation of Biotrove PCR conditions in Visual OMP 5.0 software (DNA Software Inc., Michigan, USA) estimated that the 55°C annealing temperature in the Biotrove OpenArray™ corresponded to a 60°C annealing temperature in the 7500 Real-Time PCR system.

The Biotrove OpenArray™ NT Cycler System Software (version 1.0.10.0) uses a proprietary calling algorithm that estimates the quality of each individual C_T _value by calculating a C_T _confidence value for the amplification reaction. In our assay, C_T _values with C_T _confidence values below 700 were regarded as background signals. The remaining positive amplification reactions were analyzed for amplicon specificity by studying the individual melting curves.

## Authors' contributions

RvD performed probe development, participated in the design of the study, optimized the described method, performed data acquisition, data analysis and data interpretation and drafted the manuscript.

MS participated in the design of the study, performed sequence alignments, probe development, and revised the manuscript critically.

PB participated in the design and coordination of the study, and revised the manuscript critically.

JFS participated in the probe development and provided substantial software expertise.

EO participated in optimizing the reaction conditions in the OpenArrayTM slides.

GAK provided intellectual input and revised the manuscript critically.

CDS Coordinated, supervised and contributed in the design of the study, provided intellectual input to optimize the developed method, helped to draft the manuscript and revised the manuscript critically.
